# Highly selective and active CO_2_ reduction electrocatalysts based on cobalt phthalocyanine/carbon nanotube hybrid structures

**DOI:** 10.1038/ncomms14675

**Published:** 2017-03-08

**Authors:** Xing Zhang, Zishan Wu, Xiao Zhang, Liewu Li, Yanyan Li, Haomin Xu, Xiaoxiao Li, Xiaolu Yu, Zisheng Zhang, Yongye Liang, Hailiang Wang

**Affiliations:** 1 Department of Materials Science and Engineering, South University of Science and Technology of China, Shenzhen 518055, China; 2 Department of Chemistry, Yale University, New Haven, Connecticut 06520, USA; 3 Energy Sciences Institute, Yale University, West Haven, Connecticut 06516, USA

## Abstract

Electrochemical reduction of carbon dioxide with renewable energy is a sustainable way of producing carbon-neutral fuels. However, developing active, selective and stable electrocatalysts is challenging and entails material structure design and tailoring across a range of length scales. Here we report a cobalt-phthalocyanine-based high-performance carbon dioxide reduction electrocatalyst material developed with a combined nanoscale and molecular approach. On the nanoscale, cobalt phthalocyanine (CoPc) molecules are uniformly anchored on carbon nanotubes to afford substantially increased current density, improved selectivity for carbon monoxide, and enhanced durability. On the molecular level, the catalytic performance is further enhanced by introducing cyano groups to the CoPc molecule. The resulting hybrid catalyst exhibits >95% Faradaic efficiency for carbon monoxide production in a wide potential range and extraordinary catalytic activity with a current density of 15.0 mA cm^−2^ and a turnover frequency of 4.1 s^−1^ at the overpotential of 0.52 V in a near-neutral aqueous solution.

## Abstract (web summary)

Electrochemical reduction of carbon dioxide is a sustainable way of producing carbon-neutral fuels. Here, the authors take a combined nanoscale and molecular approach to develop a highly active and selective cobalt phthalocyanine/carbon nanotube hybrid electrocatalyst for carbon dioxide reduction to carbon monoxide.

Converting carbon dioxide (CO_2_) to useful products is an attractive paradigm to mitigate the environmental problems associated with atmospheric CO_2_ concentration increase and to simultaneously benefit energy storage and chemical production^1^,^2^,^3^,^4^. Electrocatalytic CO_2_ reduction is of particular interest as it can work under ambient conditions in aqueous media and is compatible with utilization of renewable energy sources such as wind and solar energy^5^. However, the efficiency and practicality of CO_2_ electroreduction is currently hindered by the lack of cost-effective electrocatalysts with high catalytic activity, selectivity and durability^6^.

A range of materials including metals, oxides, chalcogenides, nitrogen-doped carbons and molecular complexes have been explored for catalysing CO_2_ electroreduction^7^,^8^,^9^,^10^,^11^,^12^,^13^,^14^,^15^,^16^,^17^,^18^,^19^,^20^,^21^,^22^,^23^,^24^,^25^,^26^,^27^. Among them, metal porphyrins and metal phthalocyanines constitute an attractive class of materials with distinct advantages in easy accessibility, chemical stability and structural tunability at molecular level^28^,^29^,^30^,^31^,^32^. Recently, a covalent organic framework (COF) based on cobalt-porphyrin has been reported for efficiently reducing CO_2_ to CO in aqueous electrolyte. The catalyst exhibits a Faradaic efficiency (FE) of 90% together with an optimized initial turnover frequency (TOF) as high as 3 s^−1^ at an overpotential of 0.55 V (ref. 14). In another case, iron-porphyrin derivative molecules immobilized on a carbon nanotube (CNT) electrode exhibited a TOF of 144 h^−1^ and an FE of 93% in converting CO_2_ to CO at an overpotential of 0.48 V (ref. 16). Cobalt phthalocyanine (CoPc) molecules absorbed on graphite electrode are also capable to reduce CO_2_ to CO, but the activity and selectivity are modest^13^. By modification with poly-4-vinylpridine (P4VP), the catalytic performance could be enhanced^33^,^34^. A current density of 2.0 mA cm^−2^ and a TOF of 4.8 s^−1^ with an FE of 89% for CO have been demonstrated for a CoPc-P4VP system at an overpotential of 0.64 V (ref. 34). Despite these progresses, better electrocatalyst materials are still deserved to be developed.

Here, we report a combined nanoscale and molecular approach to construct CoPc-based hybrid materials as efficient electrocatalysts for CO_2_ reduction to CO. On the nanoscale, CoPc molecules are uniformly anchored on CNTs. At an overpotential (*E*°_CO2/CO_=−0.11 V versus the reversible hydrogen electrode (versus RHE))^15^ of 0.52 V in 0.1 M KHCO_3_ aqueous solution, the CoPc/CNT hybrid catalyst shows a high and stable current density of over 10 mA cm^−2^ with a FE of over 90% for CO_2_ reduction to CO, corresponding to a TOF of 2.7 s^−1^. We find that the hybridization with CNTs significantly improves not only the catalytic activity but also the product selectivity and catalytic stability as well. The catalyst material is further upgraded with molecular level structure optimization. By introducing cyano groups to the CoPc molecular structure, we realize a superior CoPc-CN/CNT hybrid catalyst which reduces CO_2_ to CO with a TOF of 4.1 s^−1^ and a FE of 96% at an overpotential of 0.52 V, representing to the best of our knowledge the most active and selective molecular-based electrocatalyst for CO_2_ reduction to CO so far.

## Results

### Synthesis and characterization of CoPc/CNT

**Figure 1:**
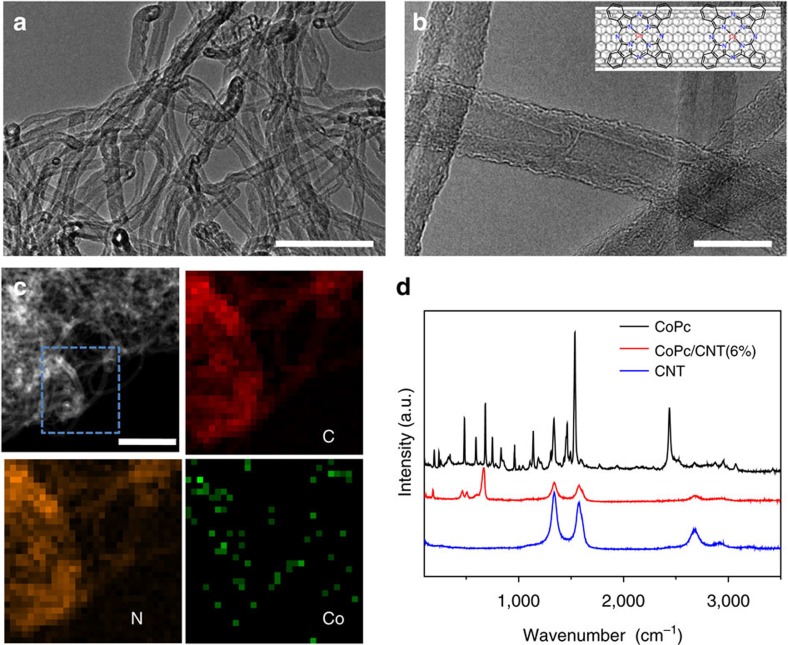
Morphological and structural characterizations of the CoPc/CNT hybrid. (**a**,**b**) TEM images of the CoPc/CNT(6%) hybrid. Inset in **b** shows a schematic representation of the CoPc/CNT hybrid. (**c**) STEM image of the CoPc/CNT(6%) material and the corresponding EDS maps of C, N and Co in the blue dash area. (**d**) Raman spectra of pure CoPc, the CoPc/CNT(6%) hybrid and pure CNTs. Scale bars, 100 nm (**a**); 20 nm (**b**); and 200 nm (**c**). EDS, energy dispersive X-ray spectroscopy; STEM, scanning transmission electron microscopy.

The CoPc/CNT hybrid was prepared by interacting CoPc and multi-walled CNTs in *N*,*N*-dimethyl formamide (DMF) with the assistance of sonication and magnetic stirring (see Methods for experimental details). DMF is a good solvent for dispersing CoPc and CNTs, allowing for effective anchoring of CoPc molecules on CNTs via strong π–π interactions^35^. Transmission electron microscopy (TEM) reveals that the morphology of the CoPc/CNT (Fig. 1a,b) resembles that of the original CNTs (Supplementary Fig. 1a,b) as nanotubular structures with an average diameter of ∼20 nm. No aggregated CoPc particles were observed. The scanning TEM image and the corresponding energy dispersive X-ray spectroscopy maps show that the distributions of C and N elements overlap and match the nanotube structures (Fig. 1c), which confirms that the CoPc molecules are uniformly dispersed on the sidewalls of the CNTs. The Co map overlaps partially with the C or N map, possibly due to the low atomic content of Co in the hybrid material. It should be pointed out that no Co signals could be detected in the original CNT sample (Supplementary Fig. 1c).

Inductively coupled plasma mass spectrometry (ICP-MS) was employed to determine the Co amount and to derive the CoPc content in the hybrid material. The Co amount was found to be 0.63 wt%, corresponding to 6.0 wt% of CoPc in the hybrid (denoted as CoPc/CNT(6%) hereafter). Raman spectroscopy was further used to characterize the CoPc/CNT hybrid (Fig. 1d). Signature vibrational peaks of CNT and CoPc can be discerned in the spectrum. It is noted that some of the CoPc vibrational features are not observed for the hybrid material, suggesting strong CoPc-CNT electronic interactions that prohibit some of the vibrational modes of the CoPc molecules on CNT. The CoPc content in the hybrid was adjusted in the range from 26 to 0.50 wt% (Supplementary Table 1). The TEM and Raman spectroscopy results of the corresponding materials are shown in Supplementary Figs 2–4. With a CoPc content of 26 wt%, wrinkled layers are clearly observed on the sidewalls of the CNTs (Supplementary Fig. 2) and the Raman spectrum shows most of the CoPc vibrational features (Supplementary Fig. 4), suggesting that CoPc aggregates have formed with such a high loading. With a CoPc loading of 2.5 wt% or lower, the CNT sidewalls appear smooth (Supplementary Fig. 3), indicating that CoPc is possibly dispersed on CNTs at molecular level.

### Electrocatalytic performance of CoPc/CNT

The catalyst materials were loaded on carbon fibre paper (CFP) substrates (catalyst loading is 0.4 mg cm^−2^ unless otherwise mentioned). Cyclic voltammetry was first performed in a phosphate buffer solution (0.2 M, pH 7.2) saturated with Ar or CO_2_ (Supplementary Fig. 5). The CoPc/CNT(6%) hybrid under Ar exhibited considerable cathodic current density at potentials <−0.35 V versus RHE, which was ascribed to hydrogen evolution reaction because hydrogen was detected as the only product with a high FE. When the solution was saturated with CO_2_, significant current increase was observed and CO_2_ reduction products were detected (Supplementary Fig. 5a). In contrast, the CFP without catalyst showed much smaller current density (Supplementary Fig. 5b). These results suggest that the CoPc/CNT hybrid has significant catalytic activity for reducing CO_2_. Control experiments further reveal that the CoPc/CNT hybrid has much higher catalytic activity than CoPc or CNTs alone (Supplementary Fig. 5a,b). CoPc/CNT hybrids with different CoPc contents were also studied (Supplementary Fig. 6). It is found that the reduction current increases with the CoPc percentage but starts to saturate when the CoPc percentage goes over 2.5 wt%. Therefore, we focus on the CoPc/CNT(2.5%) hybrid (the cobalt content is 0.26 wt%) in the following studies.

**Figure 2:**
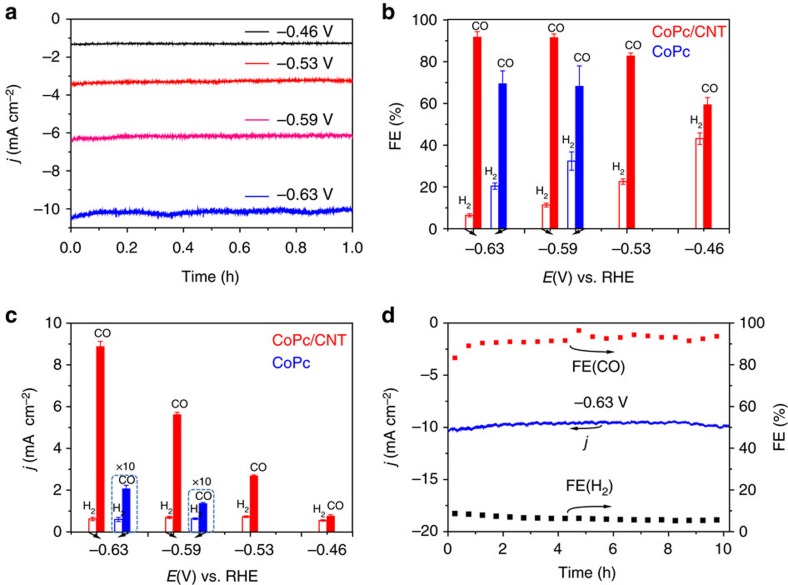
CO_2_ electroreduction catalysed by the CoPc/CNT hybrid. (**a**) Representative chronoamperograms of CO_2_ electroreduction catalysed by the CoPc/CNT(2.5%) hybrid for 1 h at various potentials in 0.1 M KHCO_3_ aqueous solution. (**b**) Faradaic efficiencies of CO_2_ reduction products in the gas phase for CoPc/CNT(2.5%) (red) and CoPc (blue) at various potentials. (**c**) Partial current densities of CO_2_ reduction products in the gas phase for CoPc/CNT(2.5%) (red) and CoPc (blue) at different potentials. The average values and error bars in (**b**,**c**) are based on six measurements during three reaction runs (two product analysis measurements were performed in each run). The error bars represent s.d. of six measurements. (**d**) Long-term stability of the CoPc/CNT(2.5%) hybrid catalyst for CO_2_ reduction operated at −0.63 V versus RHE for 10 h. The data are all *iR* corrected.

Electrochemical CO_2_ reduction in a 0.1 M KHCO_3_ aqueous solution saturated with CO_2_ (pH 6.8) was performed under controlled electrode potentials. Figure 2a shows the chronoamperograms of CoPc/CNT(2.5%) at different potentials. Little current decay (<5%) after 1 h was observed at each potential. The CoPc molecular structure remains intact over the electrolysis (Supplementary Fig. 7). A high current density of >10 mA cm^−2^ was achieved at −0.63 V versus RHE. Gas chromatography (GC) and nuclear magnetic resonance spectroscopy were used to analyse the gas and liquid products respectively. H_2_ and CO were the major gas products and no liquid products could be detected (Fig. 2b). The product distribution was found to be dependent on the applied potential. At a low potential of −0.46 V versus RHE, the FE for CO production (FE(CO)) was determined to be 59±3.4%. The FE(CO) increased with larger overpotential applied, and reached over 92% at −0.59 and −0.63 V versus RHE. In contrast, CoPc directly loaded on CFP showed significantly lower current density and faster decay (Supplementary Fig. 8). The FE(CO) was only around 68% at −0.59 and −0.63 V versus RHE (Fig. 2b). For pure CNTs, the reduction current density at −0.63 V versus RHE was smaller than 0.10 mA cm^−2^ (Supplementary Fig. 8a), and only H_2_ could be detected as the reduction product at this potential. Figure 2c shows the partial current densities of the reduction products over the CoPc/CNT(2.5%) and CoPc catalysts at various potentials. The CO production rate over the CoPc/CNT is much higher than that over the CoPc directly loaded on CFP. These results indicate that CoPc/CNT exhibits not only higher catalytic activity, but also enhanced stability and product selectivity.

A long-term operation was conducted at −0.63 V versus RHE for the CoPc/CNT catalyst. The initial current density of ∼10 mA cm^−2^ was maintained for 10 h and the FE(CO) was over 90% during the entire period (Fig. 2d), corresponding to a remarkable turnover number of 97,000 for CO_2_ conversion to CO. The quantity of CO molecules generated is ∼3,000 times more than the total number of C atoms contained in all the CoPc molecules of the CoPc/CNT catalyst. Combined with the observation that no CO or other CO_2_ reduction products are detected when either CNTs or bare CFP is used as catalyst, the result unambiguously confirms that the produced CO originates from CO_2_.

**Figure 3:**
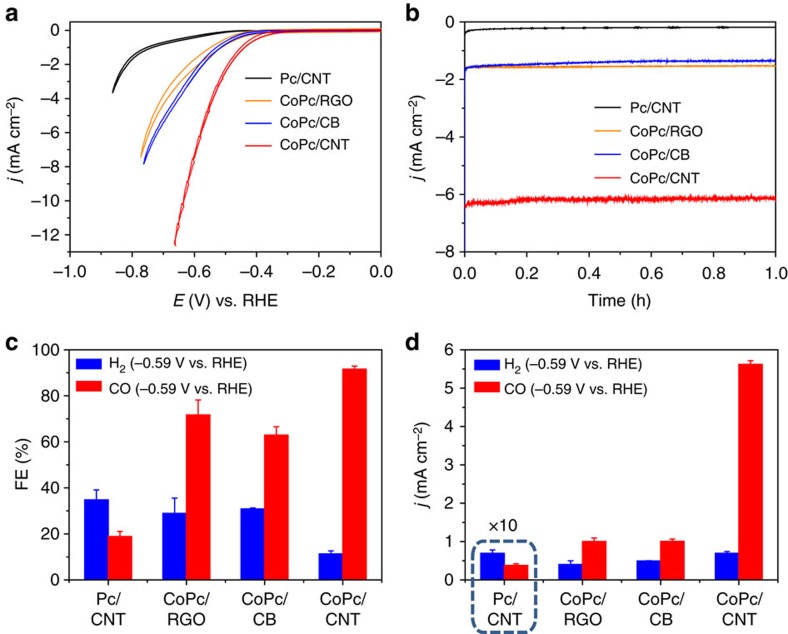
Comparison of various hybrid materials for catalysing CO_2_ electroreduction. (**a**) Cyclic voltammograms at 5 mV s^−1^, (**b**) chronoamperograms at −0.59 V versus RHE, (**c**) Faradaic efficiencies of CO_2_ reduction products, and (**d**) partial current densities of CO_2_ reduction products for Pc/CNT, CoPc/RGO and CoPc/CB in comparison with CoPc/CNT in 0.1 M KHCO_3_ solution. The average values and error bars in (**c**,**d**) are based on six measurements during three reaction runs (two product analysis measurements were performed in each run). The error bars represent s.d. of six measurements. The data are all *iR* corrected.

CoPc hybridized with other forms of nano-carbons including reduced graphene oxide (RGO) and carbon black (CB) was also studied (Supplementary Table 1). Compared with CoPc/CNT(2.5%), CoPc/RGO(2.2%) and CoPc/CB(3.3%) showed less than 1/3 of the current density at −0.59 V versus RHE with ∼10% lower FE(CO) and inferior catalytic stability (Fig. 3). The results clearly reflect the advantage of CNTs in enhancing the catalytic performance. The CNT has a higher graphitic degree than either RGO or CB and is thus likely to afford better π–π interactions with CoPc and higher electron conduction^36^. We also measured a Pc/CNT hybrid and observed much smaller reduction current density (Fig. 3b) with a much lower FE(CO) of only 19% (Fig. 3c), indicating that the Co centres in the CoPc/CNT are the catalytically active sites. The low but non-zero conversion of CO_2_ to CO on Pc/CNT is attributed to the catalytic activity of Pc itself. Recent experimental and theoretical studies have found that nitrogen dopants such as pyridinic, pyrrolic and graphitic nitrogen atoms in carbon materials can catalyse CO_2_ electroreduction to CO (refs 12, 37). Thus, it is reasonable that the nitrogen-containing Pc supported on CNTs could reduce CO_2_ to CO with certain activity.

### Cyano-substituted CoPc hybrid

**Figure 4:**
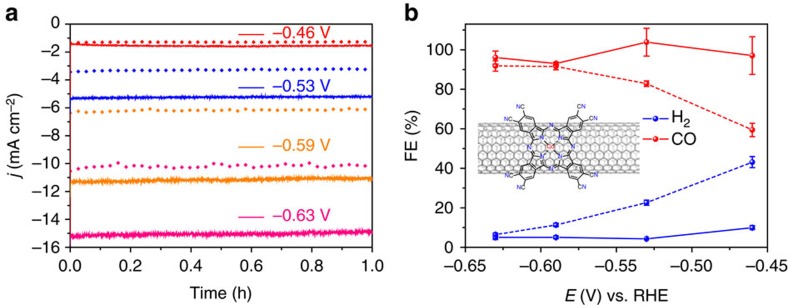
Introduction of cyano groups to CoPc enhances catalytic performance. (**a**) Chronoamperograms and (**b**) Faradaic efficiencies of reduction products at different potentials for CoPc-CN/CNT (solid line) in comparison with CoPc/CNT (dotted line). Inset in (**b**) shows the molecular structure of CoPc-CN, which is anchored on CNT. The average values and error bars in **b** are based on six measurements during three reaction runs (two product analysis measurements were performed in each run). The error bars represent s.d. of six measurements. The data are all *iR* corrected.

We further explored the potential of tuning the CoPc molecular structure for optimizing catalytic performance. Inspired by previous reports that electron-withdrawing substituents on metal phthalocyanine structures can increase the electrocatalytic performance for CO_2_ reduction to CO (refs 38, 39, 40), we synthesized cobalt-2,3,7,8,12,13,17,18-octacyano-phthalocyanine (CoPc-CN) and prepared a CoPc-CN/CNT hybrid containing 3.5 wt% of CoPc-CN (the cobalt content is 0.27 wt%, similar to that of CoPc/CNT(2.5%)) (Supplementary Fig. 9). In 0.1 M KHCO_3_, the CoPc-CN/CNT hybrid exhibits even larger reduction current density than the previous CoPc/CNT hybrid (Supplementary Fig. 10 and Fig. 4a). More impressively, higher selectivity for CO production at low overpotentials can be achieved with the CoPc-CN/CNT catalyst. The FE(CO) is already over 90% at −0.46 V versus RHE (Fig. 4b), compared with only 59% for the CoPc/CNT at the same potential. The FE(CO) maintains over 95% from −0.53 V to −0.63 V versus RHE (Fig. 4b). We also tested the CoPc-CN/CNT hybrid catalyst in 0.5 M KHCO_3_ aqueous solution. At −0.46 V versus RHE, a high current density of 5.6 mA cm^−2^ with a FE(CO) of 88% could be obtained (Supplementary Fig. 11).

## Discussion

The CoPc-CN/CNT hybrid material demonstrates outstanding catalytic performance for CO_2_ electroreduction to CO. At −0.63 V versus RHE in 0.1 M KHCO_3_, the catalyst delivers a reduction current density as high as 15.0 mA cm^−2^, with 98% of the electrons devoted to CO production. Assuming all the loaded CoPc-CN molecules are catalytically active (the electrochemically active coverage of the molecules could not be readily determined from the broad CV peaks), the TOF value for CO production is calculated to be 4.1 s^−1^, representing the lower limit of the actual TOF. The calculated TOF is slightly higher than that of other CO-selective electrocatalysts based on molecular catalytic sites (Table 1). Furthermore, our hybrid catalysts deliver much higher geometric current densities than other molecular-based catalysts under similar conditions (Table 1). At −0.46 V versus RHE in 0.5 M KHCO_3_, our CoPc-CN/CNT catalyst reaches 5.6 mA cm^−2^ with a FE(CO) of 88% (corresponding to a TOF of 1.4 s^−1^), which is already comparable to the most-active noble metal-based electrocatalysts for CO_2_ reduction to CO (Table 1). We note that the catalyst shows higher catalytic activity in 0.5 M KHCO_3_ than in 0.1 M KHCO_3_ (Supplementary Fig. 11), which is possibly due to improved mass transport of CO_2_ to the catalytic sites^41^.

A clear advantage of our CoPc/CNT and CoPc-CN/CNT hybrid materials is that they can deliver high geometrical catalytic current densities comparable to the best heterogeneous catalysts while maintaining good per-site activity comparable to the best molecular systems for CO_2_ electroreduction to CO^42^. The efficient molecule/CNT hybridization strategy allows us to realize one order of magnitude larger catalyst molecule loading (∼1.8 × 10^−8^ mol cm^−2^ for CoPc or CoPc-CN) without compromising per-molecule activity, leading to one order of magnitude increase in catalytic current density compared with the previously reported CoPc-P4VP loaded on edge-plane graphite with similar TOF^34^. For hybrid materials with higher CoPc contents, lower TOFs were expectedly observed due to aggregation of molecules (Supplementary Table 2).

The exceptional catalytic performance (activity, selectivity and durability) originates from the CNT hybridization on the nanoscale and the cyano substitution on the molecular level. The strong interactions between CoPc-CN (or CoPc) and CNTs allow for uniform distribution of the molecules on the highly conductive carbon support and thus enable a high degree of catalytic site exposure, beneficial for achieving high catalytic current densities. Rapid electron transfer from electrode to surface CoPc-CN (or CoPc) molecules anchored on CNTs facilitates fast repetitive cycling between Co(II) and Co(I) to support CO_2_ conversion to CO during the electrocatalytic process. Moreover, uniform coverage of CNTs by CoPc molecules in the CoPc/CNT catalyst material structure also minimizes exposure of carbon surface which may catalyse hydrogen evolution reaction but not CO_2_ reduction. All these contribute to the high selectivity of CO_2_ reduction over proton reduction of our hybrid catalysts^43^. Attachment to CNTs could also lower the possibility of molecule detachment from electrode and thus enhance catalytic durability.

It should be noted that our solution-phase hybridization strategy distinguishes from previous approaches where metal porphyrin or metal phthalocyanine molecules are drop-dried or dip-coated on electrodes pre-loaded with CNTs^16^,^38^. Such direct-drying methods may generate molecular aggregates, which harms catalytic site exposure and impedes efficient electron delivery from electrode to catalyst surface. To prove this concept, we used SEM to check the morphology of the CoPc loaded on CFP by drop-drying its ethanol dispersion, and observed obvious CoPc aggregates (Supplementary Fig. 12a). Replacing the ethanol with DMF is able to reduce the aggregation (Supplementary Fig. 12b), likely due to the improved CoPc solubility and higher boiling point of DMF, and thus increases the CO_2_ reduction current density (Supplementary Fig. 13). However, the catalytic performance is still substantially inferior to that of the CoPc/CNT hybrid. For the CoPc catalysts, electrons have to go through the less-conductive aggregate bulk to reach the surface molecules, which could hamper the reduction of Co(II) to Co(I). A smaller fraction of Co(I) sites on the CoPc surface and/or slower redox cycling between Co(II) and Co(I) can explain the observed lower product selectivity compared with the CoPc/CNT catalyst.

The cyano substituent on the phthalocyanine ligand is another essential contributor. The electron-withdrawing cyano groups can facilitate the formation of Co(I) which is considered as the active sites for reducing CO_2_ (ref. 44). This is supported by the more significant Co(II)/Co(I) redox transition observed at more positive potential for the CoPc-CN/CNT as compared with the CoPc/CNT (Supplementary Fig. 10b). Even though the cyano substituents may make the Co(I) sites less nucleophilic and thus bind CO_2_ less strongly, the positive shift of the Co(II)/Co(I) redox potential renders a higher fraction of Co(I) sites in the CoPc-CN/CNT catalyst than in the CoPc/CNT at low overpotentials. In the potential range (−0.46 to −0.63 V) we examined, the CoPc/CNT is only partially reduced to Co(I) (Supplementary Fig. 10b). This explains the higher current density and thus higher TOF (based on all the molecules loaded on the electrode) for the CoPc-CN/CNT hybrid catalyst. It can also be responsible for the observed higher CO selectivity for the CoPc-CN/CNT catalyst at low overpotentials. The electron-withdrawing substituents can also reduce the affinity of the cobalt centre to CO (ref. 39), which can accelerate product removal and catalytic turnover^45^. As a result, cyano substitution further enhances the catalytic performance on the basis of the CoPc/CNT hybrid material, which itself is already remarkably active and selective.

In conclusion, we have devised a combined nanoscale and molecular-level approach to construct easily accessible cobalt-phthalocyanine/CNT hybrid materials which catalyse electroreduction of CO_2_ to CO with remarkable activity, selectivity and durability in aqueous solution. The CoPc-CN/CNT shows unprecedented electrocatalytic performance, owing to the stacked effects of CNT hybridization and cyano-group substitution in the molecular structure. With the molecularly tunable phthalocyanine unit and the structurally engineerable nano-carbon support, these molecule/CNT hybrid materials represent an attractive class of electrocatalysts for converting CO_2_ emissions to sustainable fuels.

## Methods

### Chemicals

Chemicals were purchased from commercial sources and used without further purification unless otherwise noted. CoPc-CN was synthesized based on a reported method^46^. All aqueous solutions were prepared with Millipore water (18.2 MΩ cm). Organic solvents used were analytical grade. The CNTs were purchased from C-Nano (FT 9000). The purification of CNTs was done by calcining the CNTs at 500 °C in air for 5 h. After cooling down to room temperature, the CNTs were transferred into a 5 wt% HCl aqueous solution and sonicated for 30 min. The purified CNTs were collected by filtration and washed with ultrapure water for over 10 times. The quality of the CNTs was evaluated by Raman, SEM and TEM.

### Preparation of the hybrid materials

30 mg of purified CNTs were dispersed in 30 ml of DMF with the assistance of sonication for 1 h. Then, a calculated amount of CoPc or CoPc-CN dissolved in DMF was added to the CNT suspension followed by 30 min of sonication to obtain a well-mixed suspension. The mixed suspension was further stirred at room temperature for 20 h. Subsequently, the mixture was centrifuged and the precipitate was washed with DMF for three times and ethanol twice. Finally, the precipitate was lyophilized to yield the final product. Other CoPc/nano-carbon hybrids were prepared by the same method. RGO was synthesized following a previously reported method.^47^,^48^

### Material characterizations

TEM and energy dispersive X-ray spectroscopy were performed on a FEI Tecnai G2 F30 transmission electron microscope. Raman spectra were taken with Horiba LabRAM HR Evolution and Jobin Yvon LabRAM Aramis Raman spectrometers. ICP-MS was performed on an Agilent Technologies 7,700 series instrument.

### Electrochemical measurements

All electrochemical measurements were conducted using a CHI 660E Potentiostat in three-electrode configuration. Catalyst ink was prepared by dispersing 2 mg of catalyst material in a mixture of 130 μl of 0.25 wt% Nafion solution and 870 μl of ethanol with the assistance of sonication. The working electrodes were prepared by drop-drying 100 μl of catalyst ink onto carbon fibre paper (AvCarb MGL190 from Fuel Cell Store) to cover an area of 0.5 cm^2^ (loading: 0.4 mg cm^−2^). The loading of other catalysts on CFP was 0.4 mg cm^−2^ unless otherwise mentioned. The cyclic voltammetry and chronoamperometry measurements were performed in a gas-tight two-compartment electrochemical cell with a piece of glass frit as the separator (Supplementary Fig. 14). A 1 cm^2^ piece of platinum gauze was used as the counter electrode. Unless otherwise stated, the electrolyte was 0.1 or 0.5 M KHCO_3_ solution saturated with CO_2_ (pH 6.8 or 7.2). All potentials were measured against an Ag/AgCl reference electrode and converted to RHE scale based on Nernst equation. In the electrochemical measurements, *iR* corrections were made to assess the activity and selectivity of the catalyst under actual electrode potentials, so that the catalytic performance of different catalyst materials could be compared on the same bias^42^. The uncorrected potentials are listed in Supplementary Table 3. During constant-potential electrolysis, high-purity CO_2_ gas (99.999%) was delivered into the cathodic compartment at a flow rate of 5 s.c.c.m. to convey the gas products into the gas-sampling loop of a gas chromatograph (GC, SRI Instruments) for analysing the gas products. The reported TOFs and Faradaic efficiencies are average values based on three reaction runs with each containing two GC measurements (a GC measurement was initiated every 30 min). The reported cyclic voltammograms and chronoamperograms are representative data for these runs. The GC was equipped with a packed Molecular Sieve 5 A capillary column and a packed HaySep D column. Helium (99.999%) was used as the carrier gas. A helium ionization detector (HID) was used to quantify H_2_ and CO concentrations. The partial current density of CO production was calculated from the GC peak area as follows:


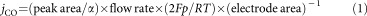



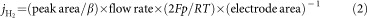


where *α* and *β* are conversion factors for CO and H_2_, respectively, determined from the calibration of the GC with standard samples, *p*=1.013 bar and *T*=293.15 K.

### Data availability

The data that support the findings of this study are available within the paper and its Supplementary Information file or are available from the corresponding authors upon request.

## Additional information

**How to cite this article:** Zhang, X. *et al*. Highly selective and active CO_2_ reduction electrocatalysts based on cobalt phthalocyanine/carbon nanotube hybrid structures. *Nat. Commun.* **8,** 14675 doi: 10.1038/ncomms14675 (2017).

**Publisher's note**: Springer Nature remains neutral with regard to jurisdictional claims in published maps and institutional affiliations.

## Supplementary Material

Supplementary Information Supplementary Figures and Supplementary Tables

## Figures and Tables

**Table 1:** Comparison of the CoPc/CNT and CoPc-CN/CNT hybrid catalysts with reported state-of-the-art high-performance CO-selective CO_2_ reduction electrocatalysts working in aqueous media.

**Catalyst**	***j* (mA cm**^−**2**^**)**	**V** **versus** **RHE**	**Electrolyte (pH)**	**Main products**	**TOF (CO) s**^−**1**^	**Ref.**
CoPc/CNT (2.5%)	∼10.0	−0.63	0.1 M KHCO_3_ (6.8)	CO (92%), H_2_ (6.4%)	2.7 (±0.0)	This study
CoPc-CN/CNT (3.5%)	∼15.0	−0.63	0.1 M KHCO_3_ (6.8)	CO (98%), H_2_ (3.3%)	4.1 (±0.1)	This study
CoPc-CN/CNT (3.5%)	∼5.6	−0.46	0.5 M KHCO_3_ (7.2)	CO (88%), H_2_ (13%)	1.4 (±0.0)	This study
Perfluorinated CoPc	∼4.4	−0.8	0.5 M KHCO_3_ (7.2)	CO(93%), H_2_ (6%)	1.6	39
CoPc-P4VP	2.0	−0.73	0.1 M NaH_2_PO_4_ (4.7)	CO (89%), H_2_ (5%)	4.8	34
COF-367-Co	3.3	−0.67	0.5 M KHCO_3_ (7.3)	CO (91%), H_2_ (20%)	0.53	14
COF-367-Co(1%)	0.45	−0.67	0.5 M KHCO_3_ (7.3)	CO (53%), H_2_ (62%)	2.6	14
CATpyr/CNT	0.24	−0.59	0.5 M KHCO_3_ (7.3)	CO (93%), H_2_ (4%)	0.04	16
FeTPP-WSCAT	∼1.0	−0.52	0.1 M KCl + 0.5 M KHCO_3_ (7.3)	CO (∼92%)	N/A	49
Au NWs	8.16	−0.35	0.5 M KHCO_3_ (7.2)	CO (94%)	0.02	50
Pd NPs	∼9.76	−0.89	0.1 M KHCO_3_ (6.8)	CO (91%)	∼0.16	19
Nanoporous Ag	∼8.7	−0.5	0.5 M KHCO_3_ (7.2)	CO (92%)	∼0.002	51

Abbreviations: CoPc, cobalt phthalocyanine; CNT, carbon nanotube; RHE, reversible hydrogen electrode; TOF, turnover frequency.
