# Supplementary material for: Highly selective and active CO2 reduction electrocatalysts based on cobalt phthalocyanine/carbon nanotube hybrid structures
Source: Nat Commun. 2017 Mar 8;8:14675. doi: 10.1038/ncomms14675 (PMC5344970; doi:10.1038/ncomms14675)
Supplement: Supplementary Information — Supplementary Figures and Supplementary Tables [file ncomms14675-s1.pdf]

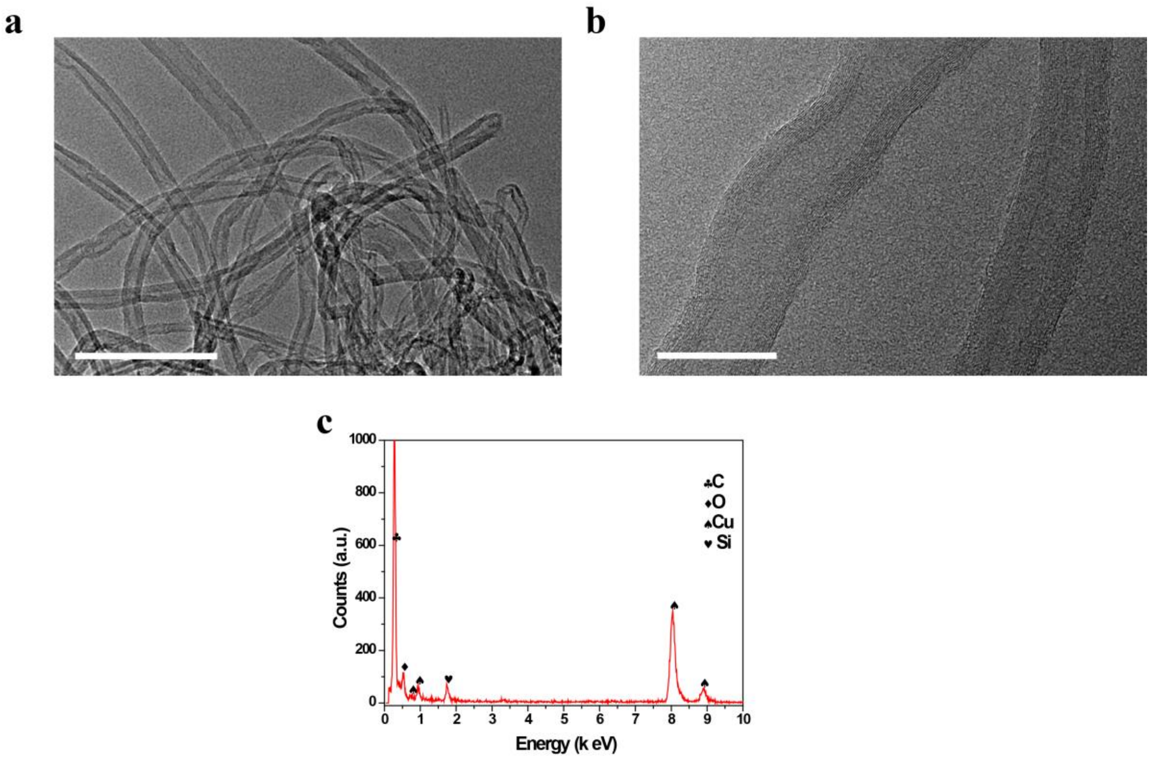

Supplementary Figure 1│ Morphology and composition of the original carbon nanotube (CNT) sample. (**a, b**) TEM images of CNT; (**c**) EDS of CNT. Cobalt is not detected in the original CNT sample (Note: The accidentally detected signal of Si is from the EDS detector). Scale bar, 100 nm (**a**); 20 nm (**b**).

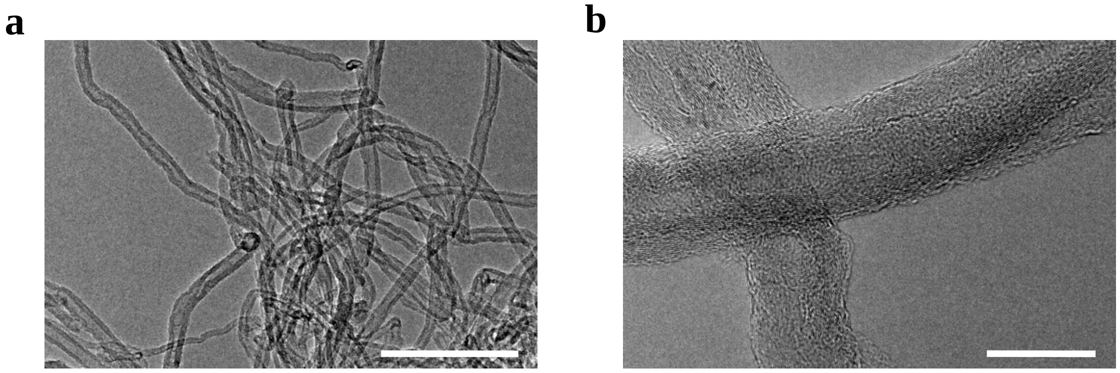

Supplementary Figure 2 │ (a, b) TEM images of the CoPc/CNT(26%) hybrid. Wrinkles are observed at the edge of nanotubes, which might correspond to the CoPc molecule aggregates. Scale bar, 100 nm (**a**); 20 nm (**b**).

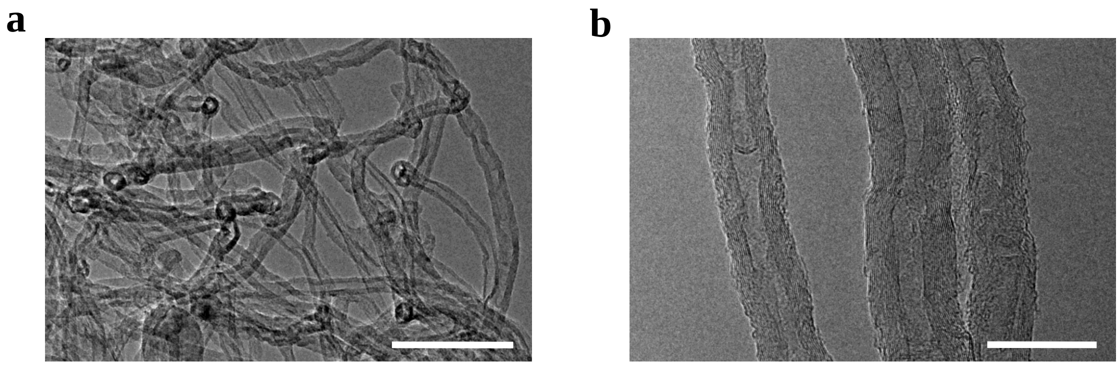

Supplementary Figure 3 | (a, b) TEM images of the CoPc/CNT(2.5%) hybrid. The CNT sidewalls appear smooth, indicating that CoPc is possibly dispersed on molecular level. Scale bar, 100 nm (**a**); 20 nm (**b**).

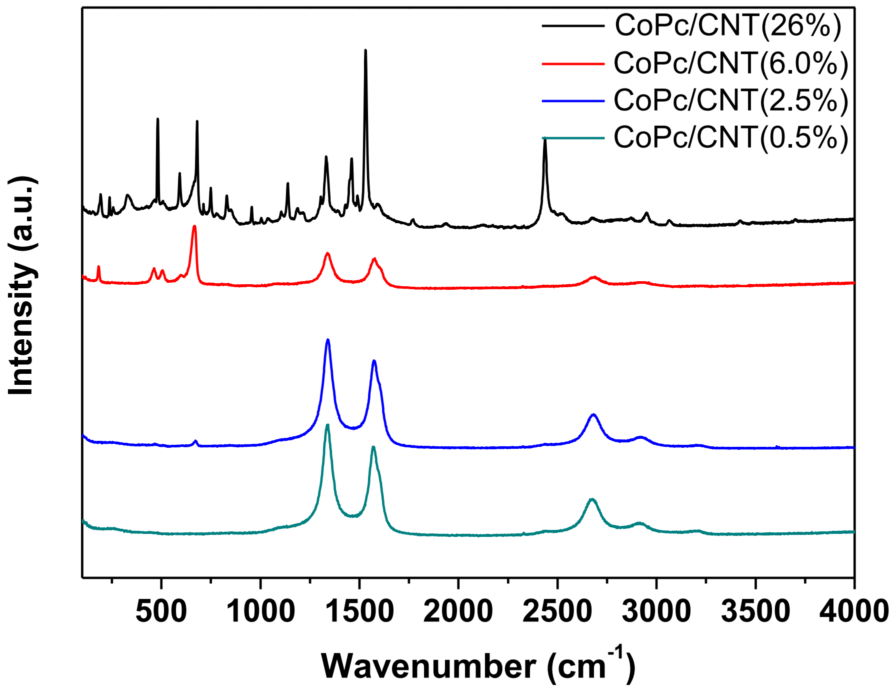

Supplementary Figure 4 │ Raman spectra of the CoPc/CNT(26%), CoPc/CNT(6%), CoPc/CNT(2.5%), and CoPc/CNT(0.5%) hybrids.

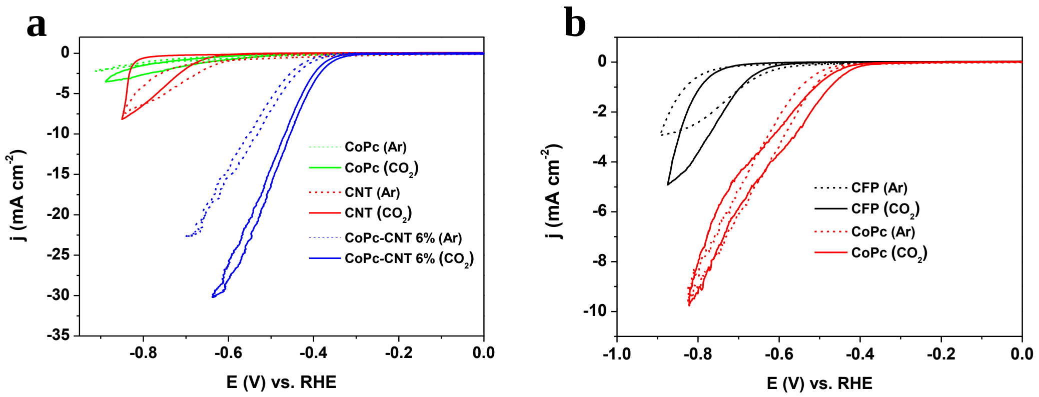

Supplementary Figure 5 | Cyclic voltammograms in 0.2 M aqueous phosphate buffer (pH 7.2). The data were recorded at a scan rate of 5 mV s^{-1} and the solutions were saturated with Ar (dashed line) or CO_2 (solid line). (**a**) CoPc/CNT(6%) hybrid catalyst, CoPc (0.02 mg cm^{-2}), and CNT. (**b**) CoPc (0.4 mg cm^{-2}) and bare carbon fiber paper (CFP) substrate. The data are all *iR* corrected.

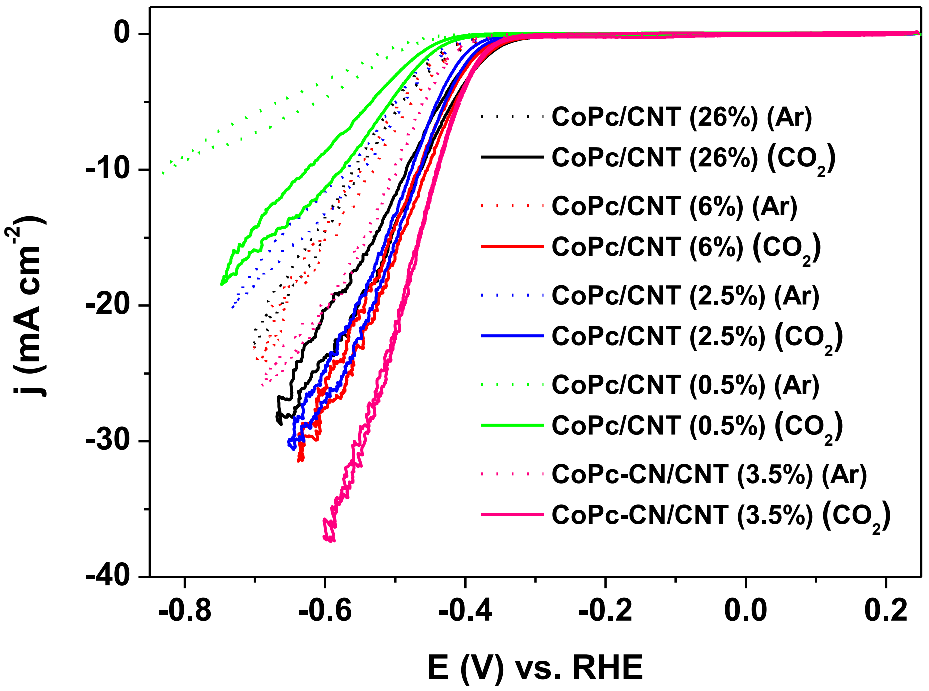

Supplementary Figure 6 │ Cyclic voltammograms of the hybrids with various content. The data were recorded in 0.2 M aqueous phosphate buffer (pH 7.2) saturated with Ar (dashed line) or CO_2 (solid line) at a scan rate of 5 mV s^{-1} for the CoPc/CNT(26%), CoPc/CNT(6%), CoPc/CNT(2.5%), CoPc/CNT(0.5%) and CoPc-CN/CNT(3.5%) hybrids. The reduction current increases with the CoPc percentage, but starts to saturate when the CoPc percentage goes over 2.5 wt%. The data are *iR* corrected.

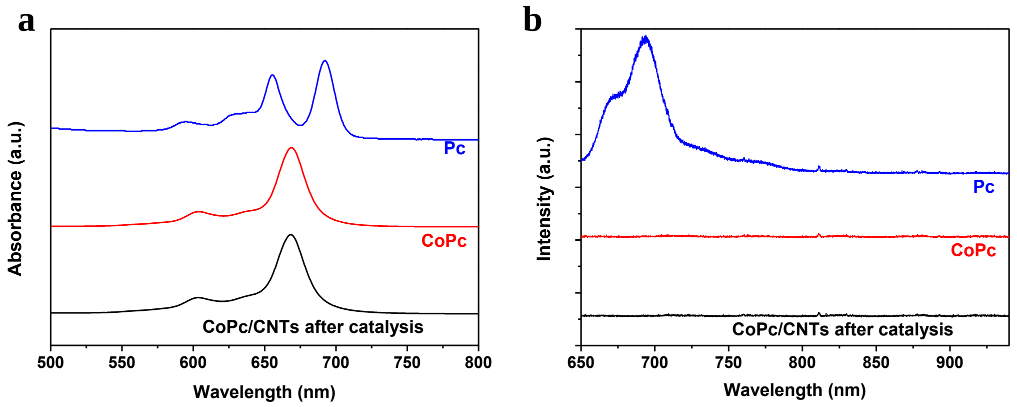

Supplementary Figure 7 | Characterizations of the hybrid after electrocatalysis. (**a**) UV-Vis absorption and (**b**) fluorescence spectra for pristine Pc, pristine CoPc, and CoPc after electrocatalyzing CO_2 reduction at -0.57 V vs. RHE after 1 h. All measurements were performed on samples dispersed in DMF. For the hybrid sample after electrolysis, the molecules were re-dissolved in DMF from the electrode by sonication.

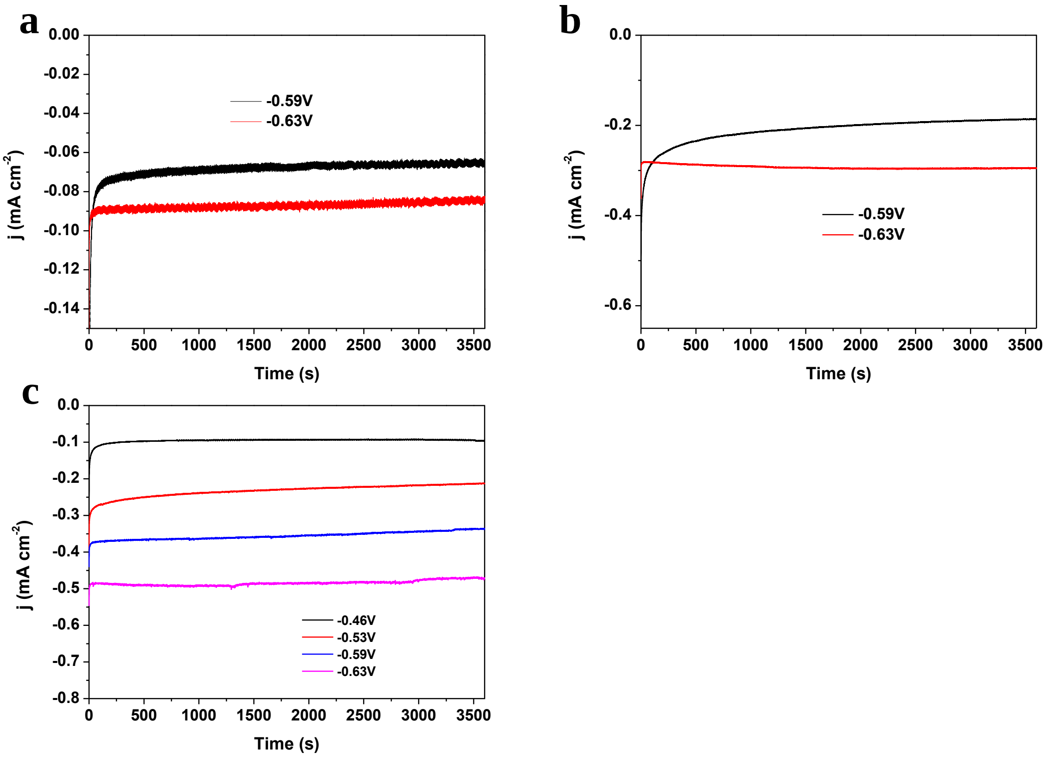

Supplementary Figure 8 | Chronoamperograms for electrochemical CO_2 reduction in CO_2 saturated 0.1 M $KHCO_3$ for 1 h at different potentials. (**a**) CNT sample. (**b**) CoPc (0.02 mg cm^{-2}). (**c**) CoPc (0.4 mg cm^{-2}).

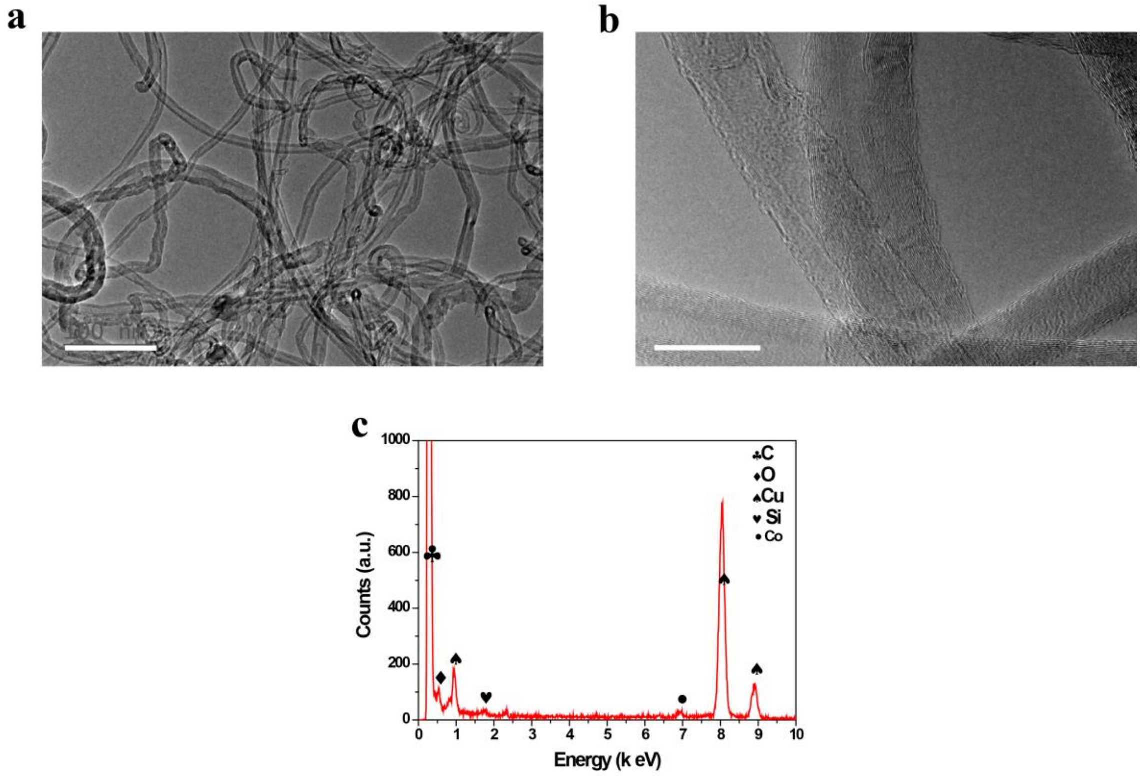

Supplementary Figure 9 │ Morphology and composition of the CoPc-CN/CNT hybrid sample. (**a, b**) TEM images of the CoPc-CN/CNT hybrid; (**c**) EDS of CoPc-CN/CNT hybrid. Co signal is detected, suggesting the existence of CoPc-CN molecules. (Note: The accidentally detected signal of Si is from the EDS detector). Scale bar, 100 nm (**a**); 20 nm (**b**).

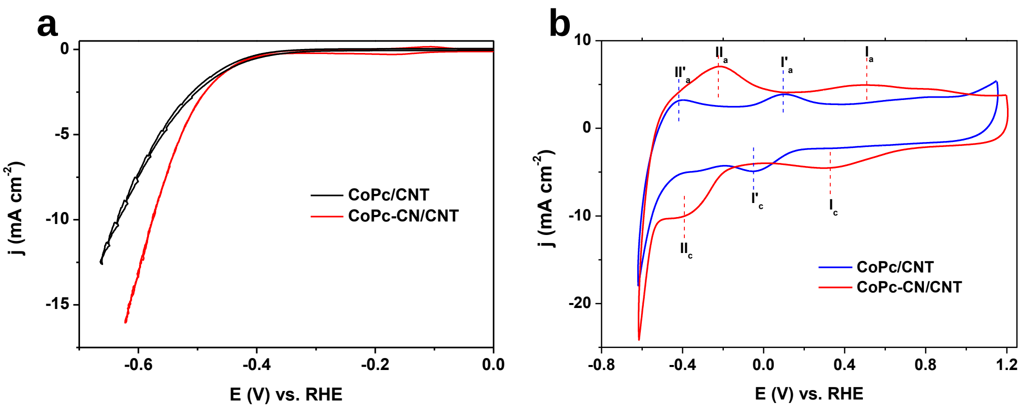

Supplementary Figure 10│ Cyclic voltammograms in 0.1 M aqueous $KHCO_3$ solution saturated with CO_2 at different scan rate. (**a**) 5 mV s^{-1} and (**b**) 400 mV s^{-1}. The Co(II)/Co(I) redox transition (II_C/II_a) is more obvious and positively shifted in CoPc-CN/CNT compared to CoPc/CNT.

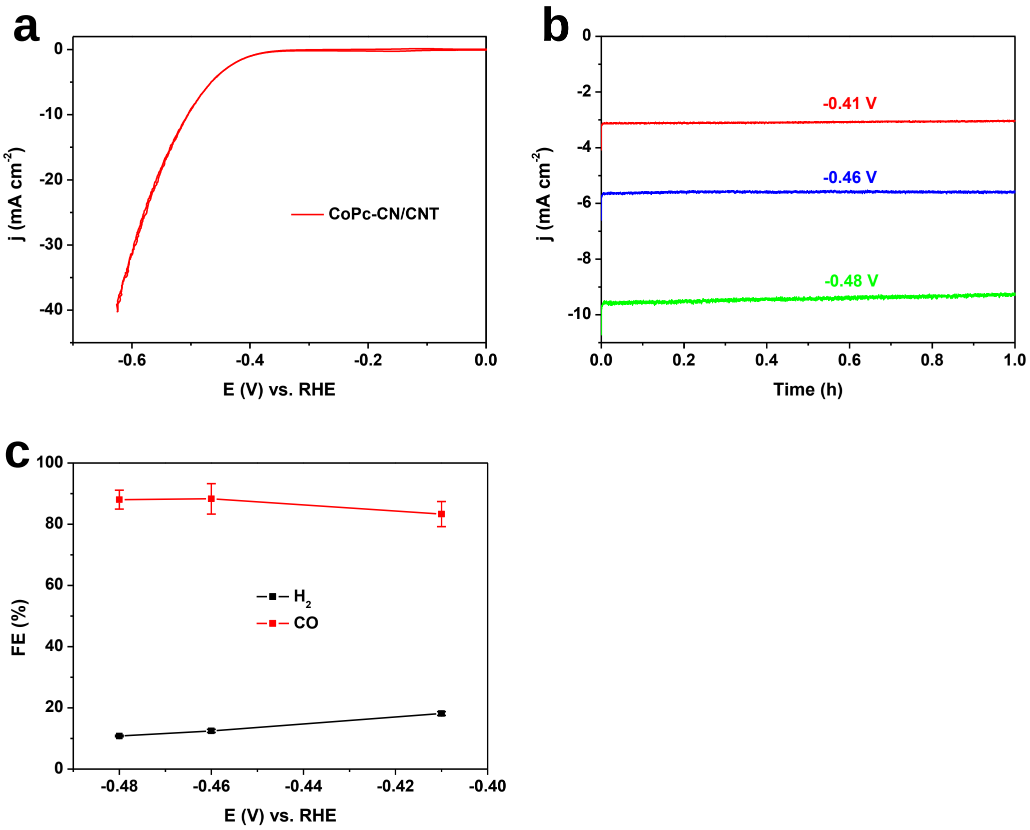

Supplementary Figure 11 | Electrocatalytic performance of CoPc-CN/CNT in 0.5 M aqueous $KHCO_3$ solution saturated with CO_2. (**a**) Cyclic voltammogram, (**b**) Chronoamperograms and (**c**) Faradaic efficiences of H_2 and CO. The average values and error bars in (**c**) are based on six measurements during three reaction runs (two product analysis measurements were performed in each run). The error bars represent S.D. of six measurements. The data are all *iR* corrected.

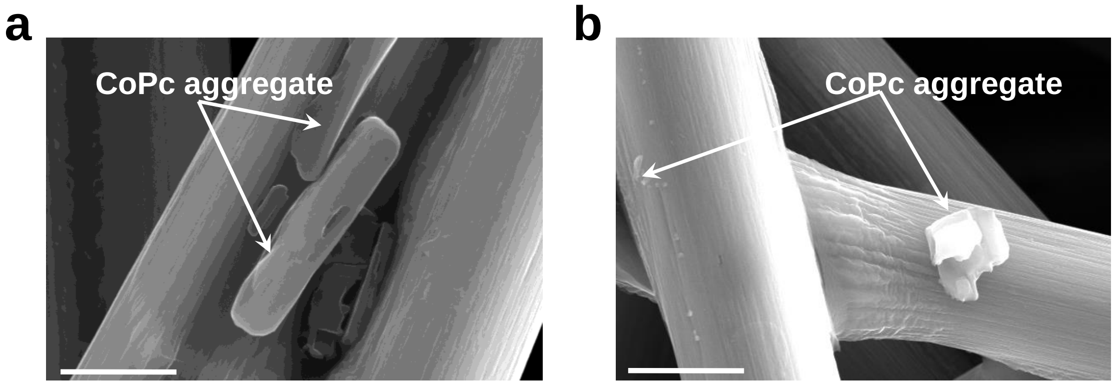

Supplementary Figure 12 │ SEM images of CoPc directly loaded on CFP. Samples prepared by drop-drying CoPc dispersed in (**a**) ethanol and (**b**) DMF. Scale bar, 4 μm (**a, b**).

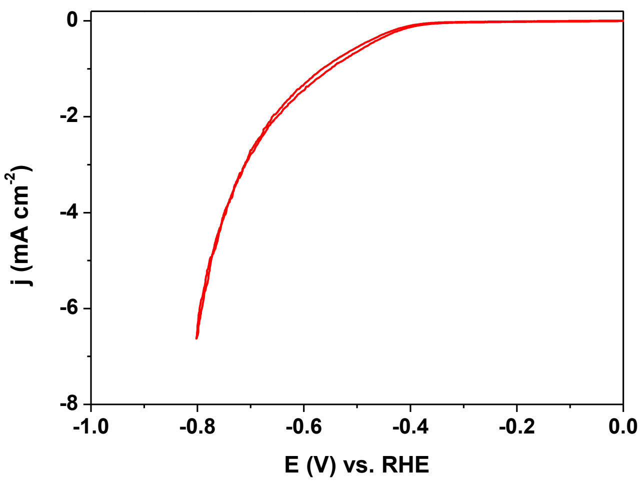

Supplementary Figure 13│ Cyclic voltammogram for CoPc directly loaded on CFP from DMF. The electrode was prepared by drop-drying from the CoPc/DMF dispersion (loading: 0.02 mg cm^{-2}). The data were recorded in 0.1 M aqueous $KHCO_3$ solution saturated with CO_2 at 5 mV s^{-1} with *iR* correction on the potential.

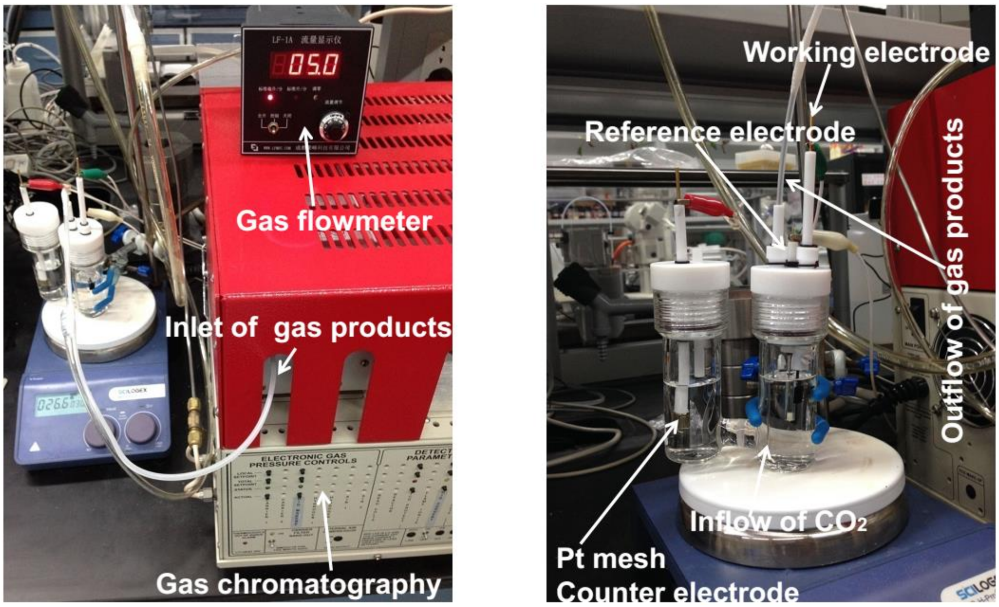

Supplementary Figure 14 | Digital photos of the electrocatalytic measurement system.

Supplementary Table 1 | Contents of phthalocyanine molecules in the hybrid catalysts determined by ICP-MS.

Initial CoPc/carbon weight ratio before mixing	Mass percentage of CoPc in the hybrid (%)	Denoted in the paper
**CoPc/CNT = 1:2**	26	CoPc/CNT(26%)
**CoPc/CNT = 1:8**	6.0	CoPc/CNT(6%)
**CoPc/CNT = 1:20**	2.5	CoPc/CNT(2.5%)
**CoPc/CNT = 1:100**	0.48	CoPc/CNT(0.5%)
**CoPc/CB = 1:8**	3.3	CoPc/CB(3.3%)
**CoPc/RGO = 1:30**	2.2	CoPc/RGO(2.2%)

Supplementary Table 2 | Calculated turnover frequency (TOF) of CO_2 reduction to CO for the electrocatalysts in CO_2-saturated 0.1 M aqueous $KHCO_3$.

Sample name	TOF(s^{-1}) at -0.63 V	TOF(s^{-1}) at -0.59 V
**CoPc-0.02**	0.031 (±0.001)	0.022 (±0.002)
**CoPc-0.4**	0.0031 (±0.0001)	0.0022 (±0.0001)
**CoPc/CNT (26%)**	0.22 (±0.00)	0.15 (±0.00)
**CoPc/CNT (6%)**	0.97 (±0.00)	0.66 (±0.00)
**CoPc/CNT (2.5%)**	2.7 (±0.0)	1.7 (±0.0)
**CoPc/CB (3%)**	0.56 (±0.01)	0.32 (±0.01)
**CoPc/RGO (2%)**	0.93 (±0.02)	0.42 (±0.02)
**CoPc-CN/CNT (3.5%)**	4.1 (±0.1)	3.0 (±0.0)

Supplementary Table 3 | Summary of catalyst electrode potentials with and without *iR* correction.

Sample	Electrolyte (pH)	Resistance (Ω)	Corrected Potentials (V)	Original Potentials (V)
**CoPc/CNT (2.5%) (Chronoamperogram, TOF)**	0.1M $KHCO_3$ (6.8)	30	-0.46<br>-0.53<br>-0.59<br>-0.63	-0.48<br>-0.58<br>-0.68<br>-0.78
**CoPc-CN/CNT (3.5%) (Chronoamperogram, CV, TOF)**	0.1M $KHCO_3$ (6.8)	46	-0.46<br>-0.53<br>-0.59<br>-0.63	-0.50<br>-0.65<br>-0.85<br>-0.97
**CoPc-CN/CNT (3.5%) (Chronoamperogram, CV, TOF)**	0.5M $KHCO_3$ (7.2)	14	-0.41<br>-0.46<br>-0.48	-0.43<br>-0.50<br>-0.55
**CoPc-0.02 mg cm^{-2} (Chronoamperogram, CV, TOF)**	0.1M $KHCO_3$ (6.8)	47	-0.63<br>-0.59	-0.64<br>-0.59
**CoPc-0.4 mg cm^{-2} (Chronoamperogram, CV, TOF)**	0.1M $KHCO_3$ (6.8)	49	-0.63<br>-0.59	-0.65<br>-0.61
**CoPc/CNT (26%) (CV, TOF)**	0.1M $KHCO_3$ (6.8)	45	-0.63<br>-0.59	-0.82<br>-0.72
**CoPc/CNT (6%) (CV, TOF)**	0.1M $KHCO_3$ (6.8)	48	-0.63<br>-0.59	-0.83<br>-0.72
**CoPc/CNT (2.5%) (CV)**	0.1M $KHCO_3$ (6.8)	40	-0.63<br>-0.59	-0.82<br>-0.73
**CoPc/CB (3%) (Chronoamperogram, CV, TOF)**	0.1M $KHCO_3$ (6.8)	47	-0.63<br>-0.59	-0.70<br>-0.63
**CoPc/RGO (2%) (Chronoamperogram, CV, TOF)**	0.1M $KHCO_3$ (6.8)	48	-0.63<br>-0.59	-0.76<br>-0.63
**CNT (Chronoamperogram, CV)**	0.1M $KHCO_3$ (6.8)	44	-0.63<br>-0.59	-0.63<br>-0.59
**Pc/CNT (Chronoamperogram, CV)**	0.1M $KHCO_3$ (6.8)	49	-0.59	-0.59
